# Cerebrovascular reserve in moyamoya disease: relation to cerebral blood flow, capillary dysfunction, oxygenation, and energy metabolism

**DOI:** 10.3389/fneur.2023.1190309

**Published:** 2023-07-20

**Authors:** Teodor Svedung Wettervik, Markus Fahlström, Johan Wikström, Anders Lewén, Per Enblad

**Affiliations:** ^1^Department of Medical Sciences, Section of Neurosurgery, Uppsala University, Uppsala, Sweden; ^2^Department of Surgical Sciences, Section of Neuroradiology, Uppsala University, Uppsala, Sweden

**Keywords:** energy metabolism, moyamoya disease, cerebrovascular circulation, cerebrovascular circulation-physiology, magnetic resonance imaging

## Abstract

**Background:**

Cerebral hemodynamics in moyamoya disease (MMD) is complex and needs further elucidation. The primary aim of the study was to determine the association of the cerebrovascular reserve (CVR) with cerebral blood flow (CBF) disturbances, oxygen extraction fraction (OEF^max^), and energy metabolism (${\text{CMRO}}_{2}^{\text{max}}$) in MMD, using arterial spin label magnetic resonance imaging (ASL-MRI) before and after acetazolamide administration.

**Methods:**

Thirty-nine ASL-MRI scans with a concurrent acetazolamide challenge from 16 MMD patients at the Uppsala University Hospital, Sweden, 2016–2021, were retrospectively analyzed. CBF was assessed before and 5, 15, and 25 min after acetazolamide administration, and the maximal response CVR^max^ was used for further analyses. Dynamic susceptibility contrast (DSC) MRI was performed 30 min after acetazolamide injection, and the data were analyzed using the Cercare Medical Neurosuite to assess capillary transit time heterogeneity (CTTH; indicating microvascular function), OEF^max^, and ${\text{CMRO}}_{2}^{\text{max}}$.

**Results:**

In the ACA territory, a lower CVR^max^ was associated with lower baseline CBF, higher CTTH, and higher OEF^max^ but not with ${\text{CMRO}}_{2}^{\text{max}}$ in generalized estimating equation models. In the MCA territory, lower CVR^max^ was associated with lower baseline CBF and higher ${\text{CMRO}}_{2}^{\text{max}}$ but not with CTTH and OEF^max.^.

**Conclusion:**

Altogether, a compromised CVR in MMD patients reflected disturbances in macro-/microvascular blood flow, oxygenation, and CMRO_2_. ASL-MRI with acetazolamide challenge is a feasible and radiation-free alternative to positron emission tomography (PET) imaging in MMD.

## Introduction

Moyamoya disease (MMD) is characterized by the progressive development of intimal hyperplasia of the terminal internal carotid artery (ICA), often together with the proximal anterior cerebral artery (ACA) and middle cerebral artery (MCA) (1). The arterial stenosis predisposes to cerebral ischemia, but cerebral autoregulation normally maintains CBF stable over a wide range of arterial blood pressure (ABP) by means of cerebral vasodilation/constriction (2). The terminal ICA stenosis then leads to an impairment of pressure autoregulation which altogether increases the susceptibility for cerebral ischemia at “normal” ABPs (1, 2). The cerebrovascular reserve (CVR) denotes the capacity to increase CBF by vasodilation at baseline ABP. Cerebral ischemia takes place when the CVR is exhausted, but increased oxygen extraction fraction (OEF) maintains adequate oxygen delivery for the cerebral metabolic rate of oxygen (CMRO_2_) to some extent (3). When the protective effects of medical therapies, e.g., blood pressure optimization, are exhausted, surgical treatment, such as direct and indirect revascularization, is an option to improve the cerebral hemodynamic status (4, 5), but the optimal time window for intervention is not fully clear (1).

Radiological imaging plays a major part in the evaluation of MMD patients to identify those at risk of neurological deterioration and acts as an aid in surgical decision-making (1), as well as follow-up after surgery. Magnetic resonance imaging (MRI) and computed tomography (CT) protocols include regular CBF measurements but usually also include assessments of the CVR by repeated CBF assessments after the administration of the cerebral vasodilator acetazolamide (ACZ). In particular, arterial spin labeling (ASL), a non-invasive and repeatable MRI-based method, to estimate CBF has shown promise and potential use in conjunction with the ACZ challenge (6, 7). In addition, positron emission tomography (PET) imaging (with ^15^O-gas) may provide further information such as OEF and CMRO_2_ (8, 9). Recently, there has been a development of perfusion-data modeling techniques, which allow for the estimation of capillary transit time heterogeneity (CTTH), OEF^max^, and ${\text{CMRO}}_{2}^{\text{max}}$, using a readily available contrast-based MRI perfusion acquisition such as dynamic susceptibility contrast (DSC) (10), which obviates the radiation associated with PET/CT (11). Particularly, capillary dysfunction, indicated by elevated CTTH, is an emerging area of interest as it may contribute to disease progression in several cerebral disorders including aneurysmal subarachnoid hemorrhage (12), carotid stenosis (13), dementias (14), and traumatic brain injury (15), but the role in MMD has so far not been investigated.

In this study, the primary aim was to determine the association of CVR with macro- and microvascular CBF disturbances, cerebral oxygenation, and energy metabolism in MMD, independently measured using ASL-MRI in conjunction with the ACZ challenge and DSC-MRI.

## Materials and methods

### Patients

All 16 patients with MMD examined with at least one MRI according to our specific MMD protocol (described below), who were managed at the Department of Neurosurgery, Uppsala University Hospital, Sweden, between 2016 and 2021 were included.

### Management protocol

The patients who had been examined with computed tomography angiography (CTA) or conventional MRI at a local hospital before admittance to our department were subsequently enrolled in our MMD protocol, which included digital subtraction angiography (DSA) and MMD-specific MRI protocol for the evaluation of hemodynamic compensation. Based on radiological findings and symptoms, patients were either followed up with our MMD-specific MRI protocol or were scheduled for revascularization surgery. The criteria for surgery were either severe symptoms with repeated ischemic attacks or progressive ischemic symptoms, ischemic lesions on MRI, or decreasing and/or low CVR, i.e., either a clear decreasing trend on repeated MRI examinations and/or CVR < 30% (7). The surgical technique used was indirect revascularization using the multiple burr-hole approach. Patients with very mild or no symptoms and stable CVR above 30% were followed with repeated clinical visits and radiological imaging.

### Magnetic resonance imaging and parameter quantification

All examinations were performed with a Philips Achieva dStream, 3.0 T (Philips Healthcare, Best, the Netherlands), using a 32-channel head-coil according to predefined protocols (6, 7). The ACZ challenge was performed on all patients (ACZ, 1 g in adults and 10 mg/kg in children). A three-dimensional (3D) pseudo-continuous ASL (pCASL) with background-suppressed gradient spin-echo read-out using a post-label delay of 2,500 ms and label duration of 1,800 ms was acquired. Acquisition duration was 5 min and 31 s, with a repetition time of 4,735 ms and echo time of 10.7 ms, spatial resolution was 3 × 3 × 6 mm^3^, and the number of slices was 14. The acquisition included two background suppression pulses, and no flow-crushing gradients were applied. The labeling plane was placed perpendicular to the brain-feeding arteries with the aid of a phase-contrast MRA survey. The 3D pCASL acquisition was performed before (baseline) and repeated 5, 15, and 25 min after the ACZ injection. ASL-based CBF maps were automatically calculated using the scanner according to the model recommended by Alsop et al. (16).

Dynamic susceptibility contrast (DSC) MRI using gradient echo, echo planar imaging (TE/TR 29/1392 ms-−1.72 × 1.72 × 5 mm^3^, the number of slices was 23) was performed 30 min after ACZ injection. Gadolinium contrast dose was 0.1 mmol/kg at 5 mL/s injection rate. DSC data were analyzed using the Cercare Medical Neurosuite (Cercare Medical, Aarhus, Denmark). Processing included motion correction and automatic detection of arterial input function.

### Parametric maps of CTTH

Parametric maps of CTTH were estimated by approximating capillary transit time distribution h (t) to a gamma distribution h (t | α, β) with shaping parameter (α) and scale parameter (β). CTTH is estimated as the standard deviation of h (t | α, β), which is equal to ($\sqrt{\alpha }\;\cdot \;\beta$). The residue function, calculated as the deconvolution between the measured concentration-time-curves and the arterial input function, is given as Mouridsen et al. (10) and Mouridsen et al. (17).


(1) $$\text{R}\left(\text{t}\right)\text{=}\int_{\text{t}}^{\infty }\text{h}\left(\text{t | }\alpha ,\;\beta \right)\text{dt}.$$

The relationship between CTTH and OEF^max^ is described in detail in Jespersen and Østergaard (18). In short, OEF^max^ is defined as follows:


(2) $$OEF^{\max}\;=\int_{0}^{\infty }h\left(t|\alpha ,\beta \right)\;Q\left(t\right)\;dt,$$

where Q (t) is a model of oxygen extraction along a single capillary as a function of time. ${\text{CMRO}}_{2}^{\text{max}}$ is defined as ${\text{CMR}{\text{O}}_{2}=C}_{\text{a}}\;\cdot \;{\text{CBF }\cdot \text{ OEF}}^{\max}$, where C_a_ is defined as arterial oxygen concentration.

Structural 3D T2-weighted fluid-attenuated inversion recovery (FLAIR) and 3D contrast-enhanced T1-weighted (CE-T1WI) images were acquired with a spatial resolution of 0.625 × 0.625 × 0.560 and 0.938 × 0.938 × 1 mm^3^ for tissue segmentation and registration purposes, respectively. Furthermore, a 3D time-of-flight MRA (spatial resolution 0.23 × 0.23 × 0.5 mm^3^) was included and used for Suzuki grade assessment if a DSA was missing.

### Data post-processing

All parametric maps including FLAIR images were registered to each patient's 3D-T1WI images and resliced to its spatial resolution using trilinear interpolation. Gray matter probability maps were derived using a multimodal approach based on the 3D-T1WI and registered FLAIR images, and GM maps were defined with a partial volume fraction above 75%. Major vascular regions, including the anterior cerebral artery (ACA), middle cerebral artery (MCA), and posterior cerebral artery (PCA), were delineated bilaterally by applying the inverse transformation from vascular MNI template space by spatial normalization of a 3D-T1WI image. All major vascular regions were resliced to the spatial resolution of the 3D-T1WI images using nearest neighbor interpolation and masked with the corresponding GM map, hence correcting for partial volume effects (6, 7, 19). All processing steps, as described above, were performed using the SPM12 toolbox (Wellcome Trust Center for Neuroimaging, London, UK). CVR was calculated as the ΔCBF_ACZ_ (CBF_post − ACZ_-CBF_pre − ACZ_) relative to CBF at baseline, performed on 5-, 15-, and 25-min data acquisitions after injection, respectively. The post-ACZ time point at which CVR had the greatest response was denoted as CVR^max^ and was included in the following statistical analysis.

### Statistical analysis

Categorical variables were described as numbers (proportions) and ordinal/continuous variables as median (range). The associations between CVR^max^ and CBF (at the baseline), CTTH, OEF^max^, and ${\text{CMRO}}_{2}^{\text{max}}$ in the ACA and MCA territories, respectively, were analyzed with a generalized estimating equation using robust standard errors that account for correlation between repeated measurements within the same patient. Derived *p*-values were two-sided, and a *p*-value below 0.05 was considered to be statistically significant.

### Ethics

All procedures performed in the studies involving humans were in accordance with the ethical standards of the institutional and national research committee and with the 1964 Declaration of Helsinki and its later amendments. Informed consent was obtained from the patients or children's parents. The study was approved by the Swedish Ethical Review Authority.

## Results

### Patients

The median age of the included 16 patients was 25 (range 8–66) years, and the female/male ratio was 13:3 (81/19%). The Suzuki grade was in median 3 (range 0–5) (Table 1). Only three patients exhibited unilateral disease (Suzuki grade 0 on the healthy side) at the time point of assessment. Eight patients had undergone previous surgery before their last MRI imaging, which included indirect revascularization in all of these cases, and one of these patients had also undergone direct revascularization.

**Table 1:** Demographics, disease stage, and treatments.

**Patients, *n* (%)**	**16 (100%)**
Age (years), median (range)	25 (8–62)
Sex (female/male), *n* (%)	13/3 (81/19%)
Suzuki grade^*^, median (range)	3 (0–5)
Previous surgery^**^	8 (50%)

^*^Only three patients exhibited unilateral disease (Suzuki grade 0 at that side). ^**^Surgery included indirect revascularization in all 8 cases and 1 of these patients had also undergone direct revascularization.

### Cerebral hemodynamics and energy metabolism—Descriptive data

**Figure 1:**
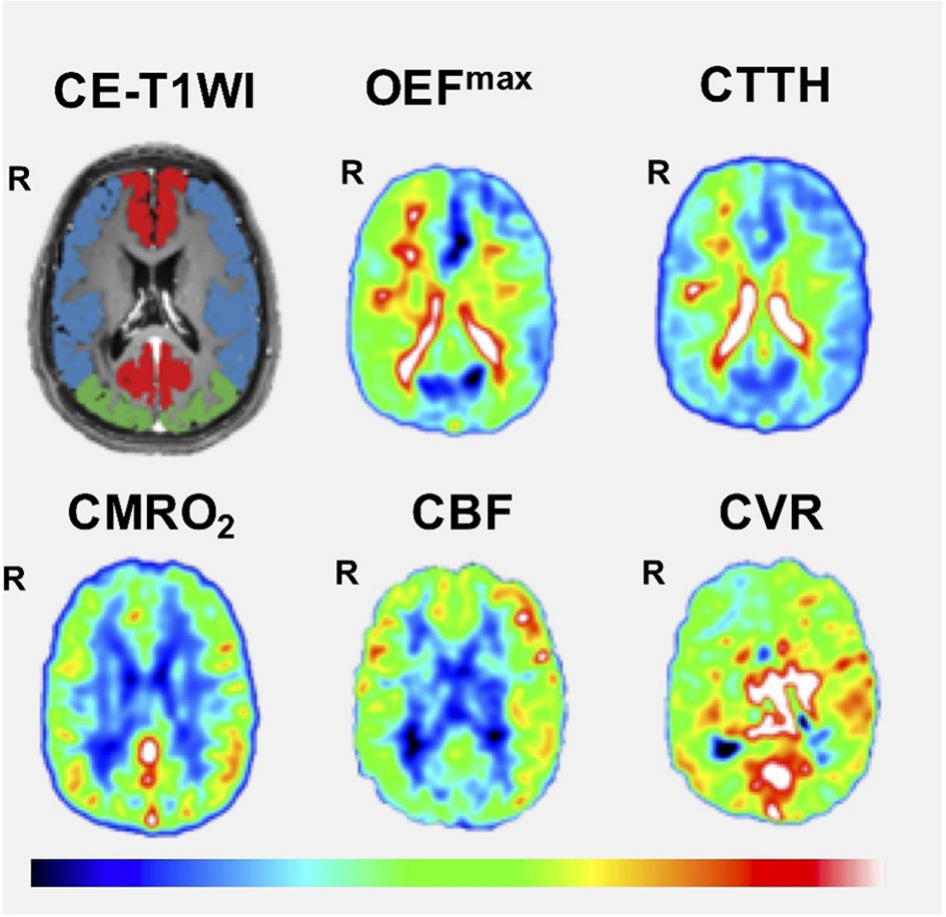
Cerebral hemodynamics using DSC-MRI as an example. The figure demonstrates one case with bilateral MMD (Suzuki grade V on the right side and I on the left side). Correspondingly, the patient exhibited a lower CVR, lower CBF, higher CTTH, and higher OEF^max^ on the right side. CE-T1WI, contrast-enhanced T1-weighted imaging; CBF, cerebral blood flow; CMRO_2_, cerebral metabolic rate of oxygen; CTTH, capillary transit time heterogeneity; CVR, cerebrovascular reserve; OEF^max^, oxygen extraction fraction.

The DSC-MRI and CVR variables in the ACA and MCA territory are described in Table 2. In the ACA territory, CBF was in median 33 (range 20–51) mL/min/100 g, CTTH in median 2.2 (range 1.3–5.3) s, OEF^max^ in median 30 (22-60) %, and ${\text{CMRO}}_{2}^{\text{max}}$ in median 4.1 (range 2.8–6.0) mL/min/100 g. In the same territory, the median CVR^max^ was 49 (range 10–95). Similar values were found in the MCA territory. Figure 1 illustrates these imaging variables in one patient.

**Table 2:** Cerebral hemodynamic and energy metabolic variables.

**Variables**	
***ACA-territory***	
CBF (mL/100 g/min), median (range)	33 (20–51)
CTTH (s), median (range)	2.2 (1.3–5.3)
OEF^max^ (%), median (range)	30 (22–60)
${\text{CMRO}}_{2}^{\text{max}}$ (mL/min/100 g), median (range)	4.1 (2.8–6.0)
CVR^max^ (%), median (range)	49 (10–95)
***MCA-territory***	
CBF (mL/min/100 g), median (range)	32 (15–59)
CTTH (s), median (range)	2.1 (1.2–6.3)
OEF^max^ (%), median (range)	31 (22–72)
CMRO_2_^max^ (mL/100 g/min), median (range)	4.0 (2.7–5.8)
CVR^max^ (%), median (range)	44 (1–85)

ACA, Anterior cerebral artery; CBF, Cerebral blood flow; ${\text{CMRO}}_{2}^{\text{max}}$, Cerebral metabolic rate of oxygen; CTTH, Capillary transit time heterogeneity; CVR^max^, Cerebrovascular reserve.

### Cerebrovascular reserve—Relation to blood flow, oxygenation, and energy metabolism

**Figure 2:**
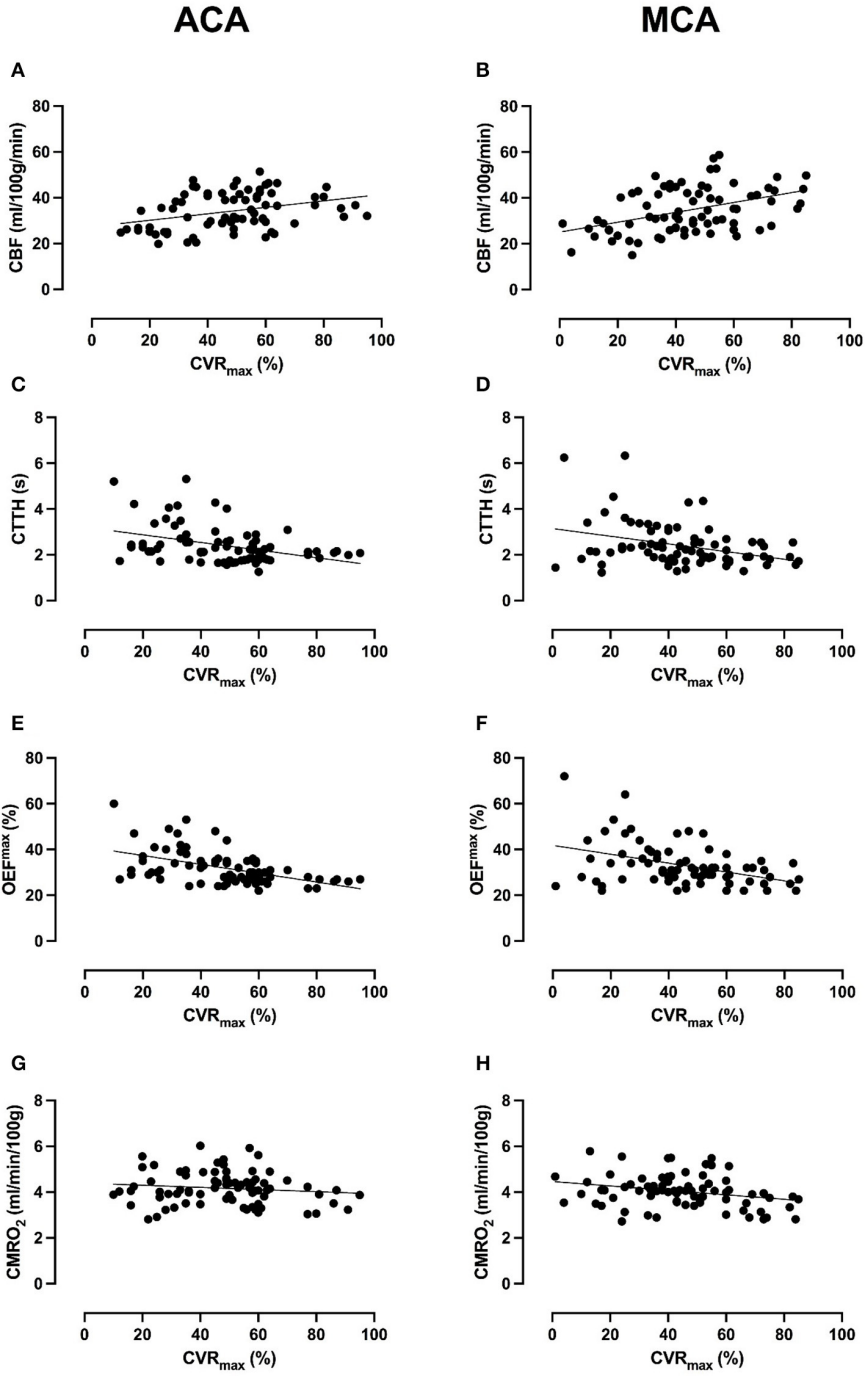
Association between CVR^max^ and cerebral hemodynamics and energy metabolism in the ACA and MCA territory. The figure demonstrates scatter plots of all 39 scans (78 ACA- and 78 MCA-territories) of the 16 MMD patients between CVR^max^ and CBF, CTTH, OEF^max^, and ${\text{CMRO}}_{2}^{\text{max}}$. Lower CVR^max^ in the ACA-territory was significantly associated with lower CBF **(A)**, higher CTTH **(C)**, and higher OEF^max^ **(E)**, but not with ${\text{CMRO}}_{2}^{\text{max}}$ **(G)** in generalized linear model analyses (Table 3). Lower CVR^max^ in the MCA-territory was significantly associated with lower CBF **(B)** and higher ${\text{CMRO}}_{2}^{\text{max}}$ **(H)**, but not with CTTH **(D)** and OEF^max^ **(F)**. Although linear trends were found, there was a quite large variability between patients and scans in CBF, CTTH, OEF^max^, and ${\text{CMRO}}_{2}^{\text{max}}$ in the lower range of CVR^max^.

In the ACA territory, lower CVR^max^ was associated with lower CBF, higher CTTH, and higher OEF^max^ but not with ${\text{CMRO}}_{2}^{\text{max}}$ (Table 3 and Figure 2). In the MCA territory, lower CVR^max^ was associated with lower CBF and higher ${\text{CMRO}}_{2}^{\text{max}}$ but not with CTTH and OEF^max^.

**Table 3:** CVR^max^ in relation to CBF, CTTH, OEF^max^, and ${\text{CMRO}}_{2}^{\text{max}}$.

			**CBF**	**CTTH**	**OEF^max^**	** ${\text{CMRO}}_{2}^{\text{max}}$ **
**CVR** ^**max**^	*ACA*	Coefficient (95%CI)	***0.141 (0.016–0.267)***	***−0.017 (−0.032–−0.001)***	***−0.193 (−0.337–−0.049)***	***–***0.005 (***–***0.013–0.004)
		*p*-value	0.027	0.036	0.008	0.297
	*MCA*	Coefficient (95%CI)	***0.215 (0.091–0.340)***	***–***0.017 (***–***0.040–0.007)	***–***0.194 (***–***0.428***–***0.040)	***−0.010 (−0.018–−0.002)***
		*p*-value	0.001	0.16	0.104	0.018

Bold and italics indicate statistical significance. ACA, Anterior cerebral artery; CBF, Cerebral blood flow; ${\text{CMRO}}_{2}^{\text{max}}$, Cerebral metabolic rate of oxygen; CTTH, Capillary transit time heterogeneity; CVR^max^, Cerebrovascular reserve.

## Discussion

This study validates the role of CVR as a predictor of CBF/microvascular and energy metabolic disturbances in MMD. It also highlights the potential role of DSC-MRI imaging for granular studies on cerebral hemodynamic and energy metabolic disturbances in MMD.

First, there was a significant association between greater CVR^max^ and higher CBF in both the ACA and MCA territories. This was not surprising considering the MMD-induced increased cerebrovascular resistance that pushes these patients near and below the lower critical threshold of autoregulation toward frank ischemia. Second, a reduced CVR^max^ particularly in the ACA territory was strongly associated with higher CTTH, indicating disturbances in microvascular blood flow. This is the first study, to the best of our knowledge, that has evaluated this in MMD. Increased CTTH by definition means a greater variation in capillary transit time and implies inefficient oxygen delivery at the tissue level (18). In healthy volunteers, functional activation of the brain leads to lower CTTH, i.e., more efficient oxygen delivery by means of capillary flow homogenization (20). In contrast, our MMD patients with a chronically compromised CVR and lower CBF did not exhibit a compensatory microvascular homogenization but rather a higher CTTH suggesting capillary dysfunction. This could both reflect functional and structural capillary disturbances. For example, transient cerebral ischemia, which is common in MMD, may induce pericyte contraction which could interfere with capillary blood flow (21). In addition, neovascularization is a structural coping mechanism for terminal ICA stenosis in MMD, and these patients typically exhibit increased cerebral microvascular density and microvessel diameter (22). This structural adaptation may well lead to a decrease in vascular resistance to augment CBF, but the corresponding microvascular flow pattern might still not be optimal for oxygen delivery. Interestingly, in atherosclerotic ICA stenosis, which is a similar but slightly different condition to MMD, it has been demonstrated that revascularization leads to early improvements in CTTH. This indicates that the capillary dysfunction was primarily functional and reversible rather than structural in that setting (13, 23). Third, a reduced CVR^max^ particularly in the ACA territory was strongly associated with increased OEF^max^. This was not surprising considering the association between low CVR^max^ and low CBF. However, although CBF was often low, oligemia/ischemia below 20 mL/min/100 g at rest only occurred in a few cases, and OEF^max^ was also increased when CBF was above this level. One explanation could be that increased oxygen extraction takes place in parallel with a gradual development of cerebral ischemia at the lower limit of autoregulation to drive further vasodilation (24). Fourth, the association between a reduced CVR^max^ with higher CMRO_2_ in the MCA territory may seem contradictory. However, the same phenomenon has been seen in atherosclerotic ICA stenosis and may reflect increased energy consumption from repeated ischemic insults and oxidative stress (25, 26), before irreversible tissue injury occurs.

**Figure 3:**
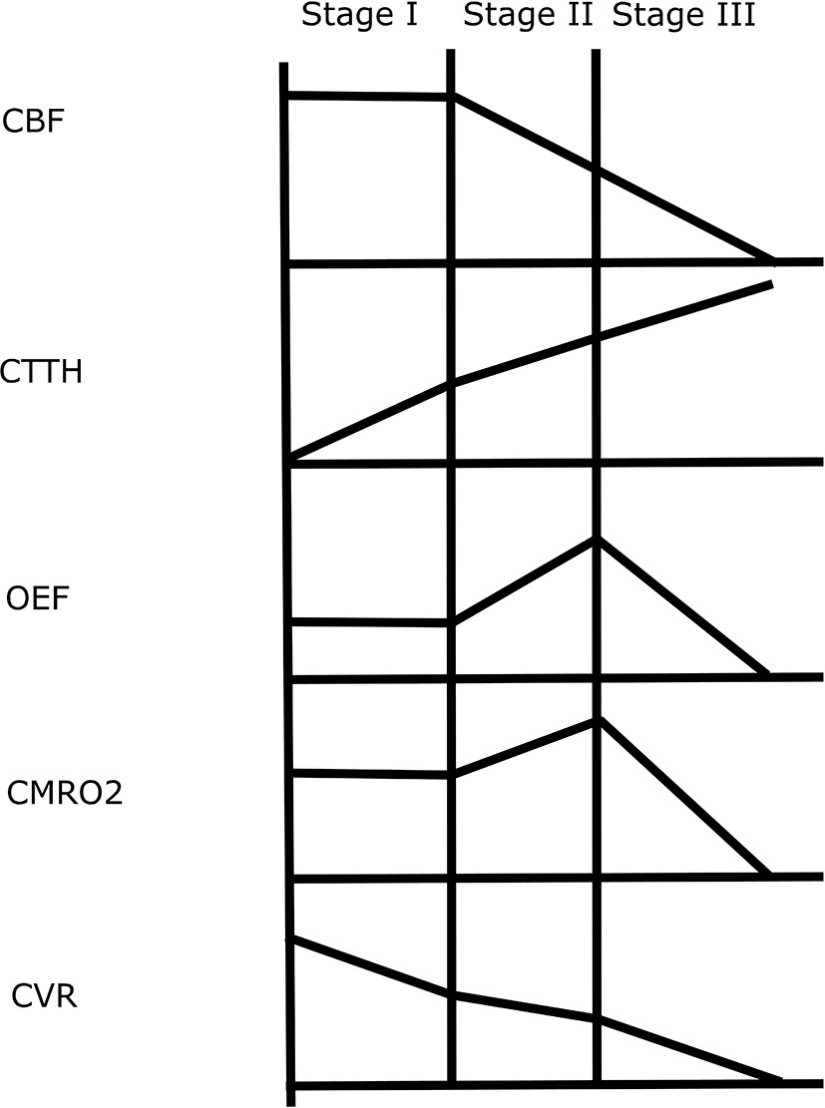
Disease progression in MMD. The figure illustrates a proposal of the cerebral hemodynamic and energy metabolic stages of MMD disease progression, a modification of the proposal of Powers et al. in ICA occlusion (26). With gradual MMD-associated terminal ICA occlusion, CBF is maintained via autoregulatory vasodilation (stage I). At the lower threshold of autoregulation, CBF deteriorates and, in parallel, OEF increases (stage II). This leads to transient cerebral ischemia/hypoxia, and CMRO_2_ is increased at this stage, probably related to oxidative stress. When the CVR becomes negative and OEF is exhausted, CMRO_2_ deteriorates and infarction takes place (stage III). However, it is important to emphasize that other mechanisms such as microthrombi and hemorrhage also contribute to stroke in MMD in addition to hemodynamic compromise due to vessel stenosis (1, 27). ACA, anterior cerebral artery; CBF, cerebral blood flow; ${\text{CMRO}}_{2}^{\text{max}}$, cerebral metabolic rate of oxygen; CTTH, capillary transit time heterogeneity; CVR, cerebrovascular reserve; ICA, internal carotid artery; MMD, moyamoya disease.

Hence, the combined picture of a reduced CVR and CBF, together with elevated CTTH and OEF, is associated with oxidative stress, thrombogenicity, and tissue damage (Figure 3) (12). This increases the susceptibility for short-term cerebral hypoperfusion in watershed areas and microembolic infarctions by turbulent blood flow as well as gradual neurodegeneration predisposing to cognitive decline and dementia in the long term (14). Future prospective studies on larger patient populations with longer follow-up periods are warranted and will be undertaken by our group on the role of CVR together with the cerebral hemodynamic and energy metabolic radiological variables on the risks of future brain infarctions as well as deterioration in neurological, cognitive, and health-related quality of life. However, it must also be mentioned that mechanisms other than hemodynamic insufficiency contribute to stroke and neurological decline in MMD, including microthrombi and intracerebral hemorrhage (1, 27).

### Methodological considerations

First, MMD is a rare disease, which limited the number of patients who were eligible for this study. Small patient cohorts are a general challenge in this research field. Nevertheless, we were able to compensate for this to some extent by optimal statistical approaches to maximize statistical power by taking into account repeated measurements and bilateral assessments of vascular territories. Second, this was a single-center study from a university hospital in Central Sweden. Furthermore, the patient cohort was relatively heterogeneous, including both pediatric and adult MMD patients, and some had undergone revascularization surgery. These factors limit the external validity and call for larger studies on more granularly defined cohorts. Third, in addition to disease progression, other variables such as pCO_2_-ventilation, hematocrit, and functional activation at the time point of imaging could have affected cerebral hemodynamic and energy metabolic variables. In future studies, we aim to assess pCO_2_ and the hematocrit before imaging to better address the impact of modulators of CBF, OEF, and CMRO_2_. Fourth, we considered assessing certain CVR^max^ thresholds for when cerebral decompensation occurs. However, although there seemed to be a gradual trend toward lower CBF and higher CTTH and OEF^max^ with lower CVR^max^, there was a quite large variability between the patients and scans (Figure 1). This indicates that a more holistic interpretation with a combination of CVR^max^, CBF, CTTH, OEF^max^, and ${\text{CMRO}}_{2}^{\text{max}}$ might be more valuable. Fifth, CTTH, OEF^max^, and ${\text{CMRO}}_{2}^{\text{max}}$ were assessed 30 min after ACZ injection. We cannot exclude that the ACZ effect still had a small effect on cerebral hemodynamics although this should be limited (28). Sixth, our study focused on estimations of CVR^max^, CBF, CTTH, OEF^max^, and ${\text{CMRO}}_{2}^{\text{max}}$ based on contrast MRI perfusion in MMD patients, but we aim to explore these associations with ASL-MRI and compare to PET imaging both in healthy controls and MMD patients in prospective trials.

## Conclusion

The study supports the validity of CVR as a predictor of disturbances in CBF, CTTH, and OEF and a compromised energy metabolic state in MMD. This is relevant for centers without a capacity for more granular cerebral hemodynamic and energy metabolic imaging. Future larger and prospective studies are warranted to determine how CVR and these other radiological cerebral hemodynamic and energy metabolic variables may be used to predict the risk for infarctions and deteriorations in motor and cognitive functions as well as quality of life. Such studies may aid in surgical decision-making for optimizing the time window of treatment. Lastly, ASL-MRI with DSC holds great promise as a feasible and safer method without radiation to follow-up MMD patients.

## Data availability statement

The datasets presented in this article are not readily available due to legal restrictions. Requests to access the datasets should be directed to TSW, teodor.svedung-wettervik@neuro.uu.se.

## Ethics statement

The studies involving human participants were reviewed and approved by Swedish Ethical Review Authority. Written informed consent to participate in this study was provided by the participants' legal guardian/next of kin.

## Author contributions

TSW: conceptualization, methodology, and writing—original draft. MF and AL: conceptualization, methodology, resources, and writing—review and editing. JW: conceptualization, methodology, and writing—review and editing. PE: conceptualization, methodology, resources, writing—review and editing, and supervision.

## References

[1] Scott RM Smith ER. Moyamoya disease and moyamoya syndrome. N Engl J Med. (2009) 360:1226–37. 10.1056/NEJMra0804622 19297575

[2] Wettervik TS Fahlström M Enblad P Lewén A. Cerebral pressure autoregulation in brain injury and disorders - a review on monitoring, management, and future directions. World Neurosurg. (2021) 158:118–31. 10.1016/j.wneu.2021.11.027 34775084

[3] Piao R Oku N Kitagawa K Imaizumi M Matsushita K Yoshikawa T. Cerebral hemodynamics and metabolism in adult moyamoya disease: comparison of angiographic collateral circulation. Ann Nucl Med. (2004) 18:115–21. 10.1007/BF02985101 15195758

[4] Rao VL Prolo LM Santoro JD Zhang M Quon JL Jin M. Acetazolamide-challenged arterial spin labeling detects augmented cerebrovascular reserve after surgery for moyamoya. Stroke. (2022) 53:1354–62. 10.1161/STROKEAHA.121.036616 34865510

[5] Cheung AH Lam AK Ho WW Tsang CP Tsang AC Lee R. Surgical outcome for moyamoya disease: clinical and perfusion computed tomography correlation. World Neurosurg. (2017) 98:81–8. 10.1016/j.wneu.2016.10.117 27810451

[6] Fahlström M Lewén A Enblad P Larsson EM Wikström J. High intravascular signal arterial transit time artifacts have negligible effects on cerebral blood flow and cerebrovascular reserve capacity measurement using single postlabel delay arterial spin-labeling in patients with moyamoya disease. AJNR. (2020) 41:430–6. 10.3174/ajnr.A6411 32115416 PMC7077908

[7] Fahlström M Wikström J Borota L Enblad P Lewén A. Variable temporal cerebral blood flow response to acetazolamide in moyamoya patients measured using arterial spin labeling. Front Neurol. (2021) 12:615017. 10.3389/fneur.2021.615017 34168605 PMC8217767

[8] Setta K Kojima D Shimada Y Yoshida J Oshida S Fujimoto K. Accuracy of brain perfusion single-photon emission computed tomography for detecting misery perfusion in adult patients with symptomatic ischemic moyamoya disease. Ann Nucl Med. (2018) 32:611–9. 10.1007/s12149-018-1283-7 30030783

[9] Kato H Shimosegawa E Oku N Kimura Y Kajimoto K Tanaka M. Cerebral hemodynamics and oxygen metabolism in patients with moyamoya syndrome associated with atherosclerotic steno-occlusive arterial lesions. Cerebrov Dis. (2008) 26:9–15. 10.1159/000135647 18511866

[10] Mouridsen K Hansen MB Østergaard L Jespersen SN. Reliable estimation of capillary transit time distributions using DSC-MRI. J Cereb Blood Flow Metab. (2014) 34:1511–21. 10.1038/jcbfm.2014.111 24938401 PMC4158667

[11] Chawla SC Federman N Zhang D Nagata K Nuthakki S McNitt-Gray M. Estimated cumulative radiation dose from PET/CT in children with malignancies: a 5-year retrospective review. Pediatr Radiol. (2010) 40:681–6. 10.1007/s00247-009-1434-z 19967534 PMC2847163

[12] Østergaard L Aamand R Karabegovic S Tietze A Blicher JU Mikkelsen IK. The role of the microcirculation in delayed cerebral ischemia and chronic degenerative changes after subarachnoid hemorrhage. J Cereb Blood Flow Metab. (2013) 33:1825–37. 10.1038/jcbfm.2013.173 24064495 PMC3851911

[13] Arsava EM Hansen MB Kaplan B Peker A Gocmen R Arat A. The effect of carotid artery stenting on capillary transit time heterogeneity in patients with carotid artery stenosis. Eur Stroke J. (2018) 3:263–71. 10.1177/2396987318772686 31008357 PMC6453199

[14] Østergaard L Jespersen SN Engedahl T Gutiérrez Jiménez E Ashkanian M Hansen MB. Capillary dysfunction: its detection and causative role in dementias and stroke. Curr Neurol Neurosci Rep. (2015) 15:37. 10.1007/s11910-015-0557-x 25956993 PMC4441906

[15] Østergaard L Engedal TS Aamand R Mikkelsen R Iversen NK Anzabi M. Capillary transit time heterogeneity and flow-metabolism coupling after traumatic brain injury. J Cereb Blood Flow Metab. (2014) 34:1585–98. 10.1038/jcbfm.2014.131 25052556 PMC4269727

[16] Alsop DC Detre JA. Multisection cerebral blood flow MR imaging with continuous arterial spin labeling. Radiology. (1998) 208:410–6. 10.1148/radiology.208.2.9680569 9680569

[17] Mouridsen K Friston K Hjort N Gyldensted L Østergaard L Kiebel S. Bayesian estimation of cerebral perfusion using a physiological model of microvasculature. Neuroimage. (2006) 33:570–9. 10.1016/j.neuroimage.2006.06.015 16971140

[18] Jespersen SN Østergaard L. The roles of cerebral blood flow, capillary transit time heterogeneity, and oxygen tension in brain oxygenation and metabolism. J Cereb Blood Flow Metab. (2012) 32:264–77. 10.1038/jcbfm.2011.153 22044867 PMC3272609

[19] Petr J Schramm G Hofheinz F Langner J van den Hoff J. Partial volume correction in arterial spin labeling using a Look-Locker sequence. Magnetic resonance in medicine. (2013) 70:1535–43. 10.1002/mrm.24601 23280559

[20] Østergaard L. Blood flow, capillary transit times, and tissue oxygenation: the centennial of capillary recruitment. J Appl. Physiol. (2020) 129:1413–21. 10.1152/japplphysiol.00537.2020 33031017

[21] Yemisci M Gursoy-Ozdemir Y Vural A Can A Topalkara K Dalkara T. Pericyte contraction induced by oxidative-nitrative stress impairs capillary reflow despite successful opening of an occluded cerebral artery. Nat Med. (2009) 15:1031–7. 10.1038/nm.2022 19718040

[22] Czabanka M Peña-Tapia P Schubert GA Woitzik J Vajkoczy P Schmiedek P. Characterization of cortical microvascularization in adult moyamoya disease. Stroke. (2008) 39:1703–9. 10.1161/STROKEAHA.107.501759 18403740

[23] Crespo Pimentel B Sedlacik J Schröder J Heinze M Østergaard L Fiehler J. Comprehensive evaluation of cerebral hemodynamics and oxygen metabolism in revascularization of asymptomatic high-grade carotid stenosis. Clin Neuroradiol. (2022) 32:163–73. 10.1007/s00062-021-01077-3 34487195 PMC8894147

[24] Kety SS Schmidt CF. The effects of altered arterial tensions of carbon dioxde and oxygen on cerebral blood flow and cerebral oxygen consumption of normal young men. J Clin Invest. (1948) 27:484–92. 10.1172/JCI101995 16695569 PMC439519

[25] Nemoto EM Yonas H Kuwabara H Pindzola RR Sashin D Meltzer CC. Identification of hemodynamic compromise by cerebrovascular reserve and oxygen extraction fraction in occlusive vascular disease. J Cereb Blood Flow Metab. (2004) 24:1081–9. 10.1097/01.WCB.0000125887.48838.37 15529008

[26] Powers WJ. Cerebral hemodynamics in ischemic cerebrovascular disease. Ann Neurol. (1991) 29:231–40. 10.1002/ana.410290302 2042939

[27] Pompsch M Veltkamp R Diehl RR Kraemer M. Microembolic signals and antiplatelet therapy in Moyamoya angiopathy. J Neurol. (2022) 269:6605–12. 10.1007/s00415-022-11323-4 36002693

[28] Eskey CJ Sanelli PC. Perfusion imaging of cerebrovascular reserve. Neuroimaging Clin N Am. (2005) 15:367–81. 10.1016/j.nic.2005.05.002 16198946

